# The social prescribing of psychosocial interventions in the treatment of addictions and substance use disorders with military veterans: a reclamation of identity and belonging

**DOI:** 10.12688/f1000research.124768.2

**Published:** 2022-09-23

**Authors:** Richard Mottershead

**Affiliations:** 1 RAK College of Nursing, Ras al Khaimah Medical and Health Sciences University, Ras Al Khaimah, United Arab Emirates

**Keywords:** social prescribing, psychosocial interventions, addictions, substance misuse, military, veterans, identity, belonging

## Abstract

Social prescribing is a way of connecting individuals to a source of support within the community to help improve their health and well-being. Social prescribing programmes are being widely promoted within the United Kingdom (UK) and United States as non-pharmaceutical interventions for those living with addiction and substance misuse needs. These needs have been exasperated by the recent COVID-19 pandemic and global economic crisis, with emerging research indicating short-term and long-term detrimental effects on physical and mental health due to substance misuse and addictions. Psychosocial interventions utilize psychological or social factors rather than an overreliance on biological interventions to treat the health impacts of mental illnesses such as addictions and substance use disorder. In this paper, I will discuss the associated determinants of addictions and substance for the military veteran population, as well as how the social prescribing of psychosocial interventions could be used to reaffirm participant’s identity and enhance their sense of belonging for military veterans, using a real-world example in Wales, UK.

## Introduction

Individuals with addictions and substance use disorders are known to face a number of challenges and needs associated with social determinants of health including medical, social, emotional, financial, legal and housing. These challenges require innovative care pathways that create solutions, in addition to evidence based treatment for substance use problems ( EMCDDA, 2016). As highlighted previously by the author ( Mottershead and Ghisoni, 2021) psychosocial interventions are crucial in supporting the recovery of military veterans. Psychosocial interventions can also be adopted through social prescribing for effective management of addiction and related issues, and can be successfully used as independent treatment methods as well as adjuncts to pharmacological treatment plans for military veterans. Public Health Wales (2018) describes social prescribing as a scheme that can involve a variety of activities which are usually provided by third sector services within the community and are accessible. These activities include but are not limited to - self-help, physical and social activities with an encouragement of hobbies that focus on the individual and not a specific illness.

The 21st Century has seen the continuation of armed conflict, exposing military personnel to the rigours of warfare and the challenges of transition back to a civilian identity. There has been a renewed realisation that there exists a sub-group of service-personnel leaving the Armed Forces with underlying mental health problems, including addictions and substance misuse disorders. These issues become apparent during the transition to a civilian life, and an overreliance and use of alcohol or substances can often become a maladaptive coping mechanism. Previously, the author ( Mottershead, 2019) has expressed concern that there was evidence that once military service personnel are discharged, there appears to be no communication to relevant health and social services to allow for rehabilitation and treatment of mental health conditions, including addictions and substance misuse disorders. Research by Fear *et al.* (2010) states that the prevalence of heavy drinking is higher with serving personnel than with their civilian counterparts. Hossain *et al.* (2020) explained that this is a concern, as following a major crisis or trauma an individual may be at an increased risk of using substances to manage stress related anxiety. Johnsen *et al.* (2008) found that for those participants identified as homeless, these participants believed that there was a link between their current alcohol abuse and the drinking culture that they had been exposed to within the Armed Forces.

The use of psychosocial interventions is not a new revelation. In 1942 in Birmingham, United Kingdom (UK) at the Northfield Military Hospital, a controversial approach was adopted that sought to rehabilitate neurotic casualties of war ( Bridger, 1985). This therapeutic community was short-lived due to its operating parameters, which were outside of the normal treatment plans of that time. This seminal work along with the previous success in the Peckham experiment in the 1930s, which saw the creation of an “unintentional therapeutic community” ( Bridger, 1985) began to establish an evidence base for a shift away from the dominant medical model. In this article, the author will present, within a review of literature, how the social prescribing of psychosocial interventions could enhance self-image and create a sense of belonging for military veterans struggling to adjust to the transition of a civilian identity.

## Alcohol and substance misuse within the veteran population

Within western nations, there appears to be a culture of reliance and overuse of alcohol within military services. This is highlighted within the research of Ames and Cunradi (2005) and Fear *et al.* (2010) within the United States and UK. This research evidence shows that alcohol consumption rates in military service personnel exceed those of civilian counterparts across all age groups. This does not appear to be a camouflaged concern in so much as to say that military leaders are aware of the negative impact that excessive drinking has on the health of their troops, their effectiveness and wider reputation of Armed Forces ( Ames and Cunradi, 2005; Iversen *et al*. 2007; Fear *et al.,* 2010).

In 2018, a report published by Combat Stress indicated that substance misuse issues were found to be higher within the veteran community than civilian counterparts. Within the 743 participants (veterans) included within the study, 8.6% had posttraumatic stress disorder (PTSD) and 57.5% had a diagnosis of another mental health problem. Alarmingly, there were higher rates of alcohol related problems (81.6%), with a lower use of illegal substances at 16.8%, and 2% prescription medications ( Ashwick and Murphy, 2018). Previously collaborative research between Help for Heroes and the Kings Centre for Military Health Research (KCMHR) noted that for those that had served within a 20-year period, there was a 10% prevalence of mental health illnesses. These illnesses included alcohol dependency and substance misuse, which would need substantial interventions from health and social care providers ( Help for Heroes & KCMHR, 2015). Recently, a qualitative research study commissioned by the Forces in Mind Trust ( Bradley *et al.*, 2021) undertook an exploration of substance misuse and service leavers as they navigate forced discharge due to failing a compulsory drug test (CDT). The authors acknowledge that there is a lack of data around drug use within the UK Armed Forces but that the US has a deeper pool to draw on due to the presence of a Health-Related Behavior Surveys. Interestingly, 65% of the sample had a pre-existing experience of substance use before joining the Armed Forces and this appears to have been a significant feature in returning to this behavior as a coping mechanism for adverse life challenging ( Bradley *et al.*, 2021). Within the same study, excessive alcohol consumption was seen as part of the culture and as long as an individual was still able to attend to their duties and refrain from activities that would be perceived as troublemaking, then excessive consumption was sanctioned and approved.

Indeed, the author’s own research and role within the Government Review on former members of the Armed Forces and the criminal justice system on behalf of the Secretary of State for Justice ( Phillips, 2014) indicated that alcohol and substance use were common features noted within the arrest profile of veterans entering the criminal justice system. The Defence Analytical Services and Advice ( DASA, 2010) explored the issue of substance misuse and found that within England and Wales, male veterans were less than 50% likely to be imprisoned for drug-related offences than that of the general population but no explanation or sample size was provided. However, Bird (2007) reported that there has been a four-fold increase in the number of veterans who are being discharged due to random sampling tests. According to Gillan (2007), the Ministry of Defence (MoD) explained that these results were significantly lower than the 7% of civilian workforce statistics, although it is unclear how they arrived at this conclusion as the source of the civilian statistics was unclear.

A crucial factor to consider is that once military service personnel are discharged, there appears to be no communication to relevant health and social services to allow for rehabilitation and treatment for the offending behaviour, which is often linked to alcohol consumption. Indeed, no information was given to be able to ascertain how many of the reported 7% were veterans. Johnsen *et al.* (2008) found that for those participants identified as homeless, these participants believed that there was a link between their current alcohol abuse and the drinking culture that they had been exposed to within the Armed Forces. In support of this argument, Fear *et al.* (2010) stated that the prevalence of heavy drinking is higher with serving personnel than with their civilian counterparts.

Fear *et al*. (2010) identifies Early Service Leavers (ESLs) as experiencing an elevated risk of suicide and heavy alcohol consumption over that of longer serving veterans. Within Wales, UK, the Health Inspectorate of Wales ( HIW, 2012) has expressed concerns that some ESLs had been discharged back into civilian life as the result of disciplinary issues, including substance misuse, without adequate liaison with statutory services and support from the MoD. There is a consensus that this group represents the most vulnerable and ineffectual at circumnavigating their transition back into civilian life especially for those exposed to combat related trauma. Kitchiner *et al.* (2012) undertook an analysis of 29 randomised controlled trials, examining the efficacy of psychosocial interventions for military veterans, and found psychosocial interventions to be beneficial for the treatment of depression and those at risk of alcohol consumption.

Exposure to armed conflict has a significant impact on those involved and can lead to PTSD. Iribarren *et al.* (2005) provides a historic account of 30 years of studies and explains that PTSD, whilst not confined to military combat, it has a higher rate for those that may have witnessed a life-threatening event or engaged in extended combat tours in warzones. Hoge *et al.* (2006) warns that anxiety and substance misuse are common comorbidities with military veterans. Similarly, Rytwinski *et al.* (2013) discusses within their research that there was a comorbidity rate of 52% with alcohol abuse being present with depression and PTSD. Sareen (2014) explains that a feature of PTSD with military veterans is the prevalence of physical health problems that are often aligned with excessive alcohol and substance misuse. Collaborating this evidence is Kessler *et al.* (1995) who notes that alcohol abuse is commonly associated with depression and PTSD within former members of the Armed Forces. The author ( Mottershead, 2019) has previously called for the need for a greater understanding of the problems faced by those who have been exposed to military culture, particularly in relation to alcohol abuse, which becomes a common feature within veteran’s life stories as they struggle with transiting to a civilian identity. The social prescribing of psychosocial interventions offers a bespoke and holistic treatment pathway to enhance resilience and develop coping mechanisms for stress, which as the literature ( Wemrell *et al.*, 2020) indicates, can lead to a dependency on addiction and misuse of substances.

## Psychosocial interventions in the reclamation of identity and belonging

A psychosocial intervention is a broad term used to describe different ways to support people to overcome challenges within their life and maintain good mental health ( O’Shea *et al.*, 2017). Psychosocial interventions have had success to aid in the normalization of the treatment process and this has relevance with military veterans who may have issues around stigma and associated shame in seeking assistance and struggling to relate to non-veteran civilian counterparts. This can be understood through Social Identity Theory ( Tajfel and Turner, 1986) which highlighted the boundaries between ‘normals’ and ‘others’. Abrams and Hogg (1990) provide further insight and explain that these theories describe a psycho-social process by which individuals categorise themselves and others into groups in order to place comparative values on themselves, thus ranking their relative position in the social hierarchy. Such ranking enables self-monitoring and potentially facilitates the reclamation of self-image. The current author ( Mottershead, 2019) has previously explained that issues around social identity are evident from his research with veterans in relation to stigma, as these individuals may strive to protect their veteran identities even if this means not mixing with non-veterans. He goes on to explain that language is important in enabling the division of individuals into categories, such as veteran and non-veteran, as the distinction can be made to ‘in’ groups and ‘out’ groups, which effectively categorises ‘us’ and ‘them’ who are divided by impenetrable boundaries that can inhibit a sense of belonging ( Mottershead, 2019).

Rather than relying on the use of medication, psychosocial interventions can be delivered through in person face-to-face as high intensity psychological therapy or in a low-intensity format via the use of Cognitive Behavioural Therapy, self-help interventions, or a combination of these support modalities ( O’Shea *et al.*, 2017). For veterans the benefits of psychosocial interventions is that they allow for a process of engaging in therapy, but with a therapeutic intervention designed around a proactive activity. This has the positive outcome of allowing the veteran to see an output for their engagement. Crucially, these psychosocial interventions can be group-based, which allows shared veteran identity to support an establishment of belonging through the familiarity of comradeship, yet empowers confidence to explore and form ownership of new identities.

This need to belong is also a feature found within Social Identity Theory, as attested by Tajfel and Turner (1986). They explain that social identity consists of “those aspects of an individual’s self-image that derive from the social categories to which he perceives himself as belonging” ( Tajfel and Turner, 1986 p.16). This inherent need to belong for military veterans could be seen to be indicative of the findings of Steger and Lopez (2011) who observed a continuing process to establish meaning through belonging. Indeed, Barron, Davies and Wiggins (2008) identify comradeship and associated societal support to be crucial in promoting a sense of belonging for military veterans. This is supported by Burnell *et al.* (2006) who cite the importance of comradeship in service personnel returning home and transitioning back to post-military identities as civilians. Indeed, the current author’s research infers that the absence of a sense of belonging can lead veterans into crime and often as a result of alcohol and substance misuse ( Mottershead, 2019). Within a more recent study ( Mottershead and Alonaizi, 2021), it was demonstrated that comradeship with fellow veterans support a reclamation of identity due to the shared life experience of a culture uniquely developed and understood by veterans within the Arabian Gulf. The results of this study have clear parallels with the UK and be understood through the use of Social Identity Theory. Additionally, this could lead to further understanding of Goffman’s (1961) mortification of self and perhaps desire to return to a pre-existing identity of veteran over other less favourable or less influential identities such as the civilian identity, which the individual may struggle to establish a sense of belonging to this transitioning identity. Consequently, the misuse of alcohol and substances become a coping mechanism within the life stories of post-military identities that fail to establish a belonging to a civilian identity.

## Future considerations

It is the intention of the author that the review of this literature will inform the development of social prescribing for existing psychosocial interventions for military veterans, reservists, emergency service personnel and their families within Wales, UK. The author believes that this care pathway will support the well-being and rehabilitation of these sub-groups, and have a positive impact on their families. This is timely as in July 2022 the Welsh Government announced a consultation on developing a framework for social prescribing. Figures in Wales indicate that from 2018 to 2021 there was an increase from 10,000 to 25,000 people benefiting from social prescribing ( Welsh Government, 2022).

This therapeutic alliance utilizes the countryside and invites the participants to join one of the regional rural hubs of the award-winning national charity - Woody’s Lodge. These rural hubs are at Ty Gwalia in North Wales, Penlan Farm in West Wales Amelia Farm in South Wales, and a recent development on Flat Holme Island within the Bristol Channel. Research by Detweiler *et al.* (2015) and Reisman (2016) supports the evidence base practice of using the countryside, gardens, greenhouses and other psychosocial interventions to facilitate relationship developments and social interaction. In addition, the sharing of real-life experiences of the challenges of combating alcohol and substance misuse will assist in developing healthy coping habits that will have a positive impact on physical and mental wellbeing ( Detweiler *et al.*, 2015). Research by Hall *et al.* (2020) and Tanagra *et al.* (2013) provides further reassurance that teamwork around a physical activity can have a positive impact on self-esteem and confidence for veterans who are transitioning to a civilian identity. This charity already has regional collaborative arrangements with NHS Trusts and Health Boards with a national collaboration with Veterans NHS Wales.

Woody’s Lodge network of rural hubs support and mentor veterans, emergency service personnel, reservists and their families in safe, quiet and informal surroundings. This is achieved through the use of the shared veteran identity as an ability to create effective veteran peer-support schemes centered around the social prescription of psychosocial interventions that built on a belonging to this shared veteran identity. This process is an aid to those living with addiction and reliance on substances, whose traditional hospital admissions have not been effective. The project is carried out by the registered UK charities Woody’s Lodge and Wintergreen UK – CIC which collectively provide the psychological and social support holistically. Both organisations offer complementary psychosocial interventions, free of charge to people with lived experience of health and social care challenges. This approach uses an evidence base to create bespoke psychosocial interventions to personalise recovery without needing further access to increased medical or other interventions ( Cipriani *et al.*, 2017).

## Conclusions

This review has highlighted an awareness of the benefits of psychosocial interventions, as a therapeutic treatment option for addictions, substance misuse and wider mental health. However, 21 ^st^ Century practices persist to treat the health needs of military veterans with predominantly pharmaceutical interventions. The author advocates for a biopsychosocial approach as realised through the care pathway of the social prescribing of psychosocial interventions. The author also advocates for a broader approach that dovetails medical prescribing alongside social prescribing so that personal health needs link to the importance of acknowledging social relationships and their impact on personal health needs of military veterans.

The author has sought to highlight that military veterans with addiction- and substance-related problems can be treated using non-medical interventions that can be socially prescribed. A real-world solution has been presented with the presentation of a national project that uses psychological treatments and interventions to support improved mental health. This paper acknowledges the strength of the military identity and how transitional challenges can create an entrenched social identity, which can inhibit a sense of belonging to new roles and identities within civilian life. Through the proposed use of veteran peer-support structured around psychosocial interventions, the rural hubs of Woody’s Lodge will empower veterans and their families through the adoption of the proposed therapeutic framework. The use of psychosocial interventions via the rural hubs has the potential to become a fully integrated pathway for primary care and social prescribing practices in Wales for the veteran community. This integrated approach between healthcare providers, community, voluntary and statutory services means that there will be opportunities for cost-saving as well as multidisciplinary collaboration and cooperation around addictions with military veterans. Given the degrading economic climate within the UK and post-pandemic fallout it is likely that there will be a detrimental effect on veterans’ mental health.

This paper seeks to present alternatives to the traditional use of pharmaceutical treatments and how the additional use of psychosocial interventions through social prescribing will allow for an early intervention and support the rehabilitation of addictions for military veterans. The addition of social prescribing arms the health profession with an additional asset via access to community-based treatments and improved access to psychosocial therapies. The author advocates for interventions that seek to address the wider determinants of impoverished self-image and to create an awareness that embedded within veterans is a need to belong. A sense of belonging creates a positive self-image, which can lead to the development of a new civilian identity and a rewarding life.

## Data availability

No data are associated with this article.
