# Supplementary material for: The social prescribing of psychosocial interventions in the treatment of addictions and substance use disorders with military veterans: a reclamation of identity and belonging
Source: F1000Res. 2022 Sep 23;11:944. Originally published 2022 Aug 17. [Version 2] doi: 10.12688/f1000research.124768.2 (PMC9513413; doi:10.12688/f1000research.124768.2)
Supplement: Peer review history

# Peer review: Reviewer response for version 2

**DOI:** 10.5256/f1000research.138313.r151295

**Published:** 2022-09-26

**Authors:** Professor Matt Fossey

**Affiliations:** 1 Veterans and Families Institute, Faculty of Health, Social Care and Education, Anglia Ruskin University, Chelmsford, UK

Thank you for giving me the opportunity to review the revised version of this paper. The author has addressed the primary points raised in my original review. This paper now presents more of an authoritative account of the use of psychosocial interventions with veterans with addictions and substance use disorders. The Welsh example of best practice in this field offers an important opportunity for further research into the outcomes for veterans who use the service and also an exploration of how services using a similar model could be utilised for other groups who have been through challenging transitions e.g. care leavers etc.

Is the review written in accessible language?

Yes

Are all factual statements correct and adequately supported by citations?

Partly

Are the conclusions drawn appropriate in the context of the current research literature?

Partly

Is the topic of the review discussed comprehensively in the context of the current literature?

Partly

Reviewer Expertise:

I am a social scientist with an extensive portfolio of research with the military, veterans and their families

I confirm that I have read this submission and believe that I have an appropriate level of expertise to confirm that it is of an acceptable scientific standard.

# Peer review: Reviewer response for version 1

**DOI:** 10.5256/f1000research.136996.r147820

**Published:** 2022-09-02

**Authors:** Professor Matt Fossey

**Affiliations:** 1 Veterans and Families Institute, Faculty of Health, Social Care and Education, Anglia Ruskin University, Chelmsford, UK

Thank you for giving me the opportunity to review this paper. The subject matter is of great importance as the NHS seeks to increase and improve its psycho-social healthcare provision for veterans with the emergence of the Op Courage programme.

 The debate in this article is stilted somewhat by the tight word-limit, which is a shame, as the author has quite a lot to say about a significant topic and this is a challenge within this restriction.

 I believe that this article is suitable for publication with a number of changes, that I will outline below. I hope that these changes will not take away from the substance of the argument that the author is trying to make, rather enhance it:

1. It would be helpful to give a clearer definition of social prescribing -it’s worth looking at some of the excellent material developed by the Kings Fund What is social prescribing? | The King's Fund (kingsfund.org.uk). However, this paper does not really focus on “social prescribing” per se, rather the use of psychosocial interventions. Consequently, the author may wish to drop the term “social” from the title. This, I believe, will help to better indicate the focus of the paper.
2. There needs to be consistency throughout the article – where the author is referring to research on PTSD from the USA, this needs to be made clear. It is quite a challenge to use this when formulating an argument for service improvement in the UK as the numbers of patients with PTSD are not comparable. I would urge the author to use UK-based data only.
3. Issues relating to the challenges associated with military transition and substance abuse in the UK have been investigated in research funded by the Forces in Mind Trust 20210322-Galahad-Fall-Out-Briefing-Report-FINAL.pdf (pcdn.co) – this is the most up-to-date account in the UK and should be referenced – the figures within this report will be slightly more up-to-date than the Combat Stress ones cited.
4. The author needs to also consider the UK’s MOD attempts to engage with and provide support for more challenging SLs – please refer to JSP100 and the formation of the Defence Transition Service (DTS). It is also worth noting that the reduced provision for CDT failures was removed over 12 months ago – they are now entitled to support that is commensurate with their length of service.
5. As the author is proposing work with colleagues in Wales, it may also be worthwhile citing some of the work that currently being undertaken within the Principality and how the author would:
  - See this link to the existing mental health provision for veterans
  - And outline how this service could be evaluated within the context of different models  
6. Finally, it would be helpful for the flow of the article if *Woody’s Lodge *and the nature and importance of their work were introduced at the start. The argument relates to the potential of this rural retreat for the treatment of veterans and their families and I think should have more prominence earlier.

Is the review written in accessible language?

Yes

Are all factual statements correct and adequately supported by citations?

Partly

Are the conclusions drawn appropriate in the context of the current research literature?

Partly

Is the topic of the review discussed comprehensively in the context of the current literature?

Partly

Reviewer Expertise:

I am a social scientist with an extensive portfolio of research with the military, veterans and their families

I confirm that I have read this submission and believe that I have an appropriate level of expertise to confirm that it is of an acceptable scientific standard, however I have significant reservations, as outlined above.

# Response

**Published:** 2022-09-05

**Authors:** Dr. Richard Mottershead

**Affiliations:** Ras Al Khaimah Medical and Health Sciences University, United Arab Emirates

1. Response – Thank you for this suggestion and I have sought to include within the introduction a definition from a relevant document pertinent to social prescribing within the locality of the proposed initiative which has a relatable evidence base. I have not included Op Courage as this is a recent initiative within England and instead I have focused on initiatives within the country of Wales.
2. Response - The articles abstract identifies a wider scope than just a UK perspective in highlights the inclusion of US data. I acknowledge my esteemed colleagues point regarding variation in numbers between UK and US. However, the presence of PTSD and most importantly the transatlantic evidence of the beneficial use of social prescribing of psychosocial interventions is a joint objective of the author and the journal to reach a wider demographic diversity.
3. Response – I have included the relevant research study within my article which is strengthened by your suggestion. Thank you.
4. Response – As you so correctly identity the allocated word limit within the article means that a fuller exploration of all suggested points becomes restricted. As the article indicates, this is the 2 ^nd^ publication of a series and your points will be included with the subsequent publications.  
5. Response – the article highlights research and work being undertaken within Wales and I have sought to highlight this work by listing some collaborative work with additional psychological therapies services. I hope to publish a third article that will focus on the points you raise once the national rural hub network is established at the end of this year.
6. Response – it was felt by myself and the editorial team that the presentation and discussion of the topic (theory) and subsequently the presentation of the charity (practice) would guide the reader to the importance and relevance to the proposed initiative.

Overall response – I would like to thank Professor Matt Fossey for his review of my article. I acknowledge him to be one of the UK’s most highly regarded academics within the area of veteran affairs who has a genuine and heartfelt compassion to support military veterans and their families. My article has been enhanced through his review and guidance. I have submitted an updated version of my article which will be available shortly - Thank you.

 Dr. Richard Mottershead  

# Peer review: Reviewer response for version 1

**DOI:** 10.5256/f1000research.136996.r147821

**Published:** 2022-08-26

**Authors:** Professor David Taylor

**Affiliations:** 1 Gulf Medical University, Ajman, United Arab Emirates

This is a clear review of the literature surrounding the support needed for military veterans with addiction/drug-related problems. They reach the conclusion that specific psychosocial models of care should dovetail with the existing pharmaceutical approaches. 

 From what the author writes, the strength of the approach stems from the potential for psychosocial support to help the veterans repair their impoverished self-image and through that regain an identity within society as a whole. 

 This review is well-researched, grounded in a clear understanding of the participants and the literature, and is to be highly commended.

Is the review written in accessible language?

Yes

Are all factual statements correct and adequately supported by citations?

Yes

Are the conclusions drawn appropriate in the context of the current research literature?

Yes

Is the topic of the review discussed comprehensively in the context of the current literature?

Yes

Reviewer Expertise:

Qualitative research particularly in education

I confirm that I have read this submission and believe that I have an appropriate level of expertise to confirm that it is of an acceptable scientific standard.

# Response

**Published:** 2022-09-05

**Authors:** Dr. Richard Mottershead

**Affiliations:** Ras Al Khaimah Medical and Health Sciences University, United Arab Emirates

I would like to take this opportunity to thank Professor David Taylor for taking the time to review my article and acknowledging the importance and relevance of the content in supporting and treating military service personnel. His insight and guidance will allow me to write and publish with clarity and focus. 

 Thank you 

 Dr. Richard Mottershead
